# Supplementary material for: Asymmetric Henry Reaction of 2-Acylpyridine N-Oxides Catalyzed by a Ni-Aminophenol Sulfonamide Complex: An Unexpected Mononuclear Catalyst
Source: Molecules. 2019 Apr 14;24(8):1471. doi: 10.3390/molecules24081471 (PMC6514737; doi:10.3390/molecules24081471)
Supplement: Supplementary file 1 [file molecules-24-01471-s001.pdf]

Supplementary Materials for:

Asymmetric Henry Reaction of 2-Acylpyridine *N*-Oxides Catalyzed by a Ni-Aminophenol Sulfonamide Complex: An Unexpected Mononuclear Catalyst

Mouxiong Liu †, Dongdong Gui †, Ping Deng and Hui Zhou *

School of Pharmaceutical Science, Chongqing Medical University, Chongqing 400016, P. R. China; mouxiongliu@163.com (M.L.); g420646114@163.com (D.G.); 100865@cqmu.edu.cn (P.D.)

* Correspondence: hzhou@cqmu.edu.cn

† These authors contributed equally to this work.

Contents

1. The characterization of the new ligands 2

2. Other optimizations of the Henry reaction 3

3. ESI-MS analysis of the mixture of Ni/**L2**/**1a** 5

4. HPLC chromatograms for ee determination 8

5. Copies of NMR spectra 17

1. The characterization of the new ligands

L1–L10 were prepared according to the literature.[1] **L11** was synthesized by the analogy of **L1**. **L11** White solid, ^{1}H NMR (600 MHz, $CDCl_3$) δ 7.35(d, 4H, *J* = 6.4), 7.14-7.13(m, 4H), 7.04-6.92(m, 14H), 6.87(d, 4H, *J* = 7.0), 6.81(s, 2H), 6.48(brs, 2H), 4.29(d, 2H, *J* = 7.9), 3.76(s, 2H), 3.60(d, 2H, *J* = 13.1), 3.40-3.37(m, 5H), 2.26(s, 9H). ^{13}C NMR (150 MHz, $CDCl_3$) δ 153.8, 141.6, 138.1, 137.1, 136.0, 132.7, 131.4, 129.1, 128.0, 127.2, 126.84, 126.81, 126.44, 126.09, 126.04, 66.6, 62.5, 60.1, 45.8, 20.4, 19.8. ESI-HRMS calcd for $[C_{52}H_{55}N_4O_5S_2]^+$ $[M + H]^+$: 879.3608, Found 879.3603.

1. Zhang, S.; Deng, P.; Zhou, J.; Liu, M.; Liang, G.; Xiong, Y.; Zhou, H. *Chem. Commun*. **2017**, *53*, 12914-12917.

2. Other optimizations of the Henry reaction

Table S1. Screening of central metal ions in the asymmetric Henry reaction. [a]

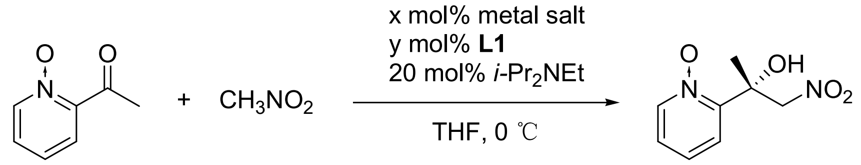

Entry	Metal salt (x)	y	x/y	Yield (%)[b]	*ee* (%)[c]
1	$Ni(OAc)_2$ (20)	10	2/1	94	76
2	$Fe(OAc)_2$ (20)	10	2/1	trace	-
3	$Co(OAc)_2$ (20)	10	2/1	34	0
4	$Cu(OAc)_2$ (20)	10	2/1	25	20[d]
5	CuOAc (20)	10	2/1	13	22[d]
6	$Zn(OAc)_2$ (20)	10	2/1	trace	-
7	$Ni(OAc)_2$ (10)	10	1/1	91	81
8	$Ni(OAc)_2$ (15)	10	1.5/1	98	81
9	$Ni(OAc)_2$ (10)	20	1/2	73	83
10	$Ni(OAc)_2$ (10)	15	1/1.5	72	84
11	$Ni(OAc)_2$ (10)	12	1/1.2	79	83
12	$Ni(OAc)_2$ (10)	11	1/1.1	86	85
13	$Fe(OAc)_2$ (10)	11	1/1.1	trace	-
14	$Co(OAc)_2$ (10)	11	1/1.1	trace	-
15	$Cu(OAc)_2$ (10)	11	1/1.1	trace	-
16	CuOAc (10)	11	1/1.1	trace	-
17	$Zn(OAc)_2$ (10)	11	1/1.1	trace	-
18	$Ni(acac)_2$ (10)	11	1/1.1	48	57
19	$Ni(OTs)_2$ (10)	11	1/1.1	86	71
20	$NiCl_2$ (10)	11	1/1.1	83	80
21	$Ni(OTf)_2$ (10)	11	1/1.1	90	83
22	$Ni(ClO_4)_2$ (10)	11	1/1.1	99	63

[a] Reactions were carried out with 2-acylpyridine *N*-oxides (0.2 mmol) with *i*-Pr_2NEt (20 mol%) in a mixture of THF (0.8 mL) and CH_3NO_2 (0.2 mL) for 20 h. [b] Isolated yield. [c] Determined by chiral HPLC. [d] The absolute configuration of the major product was inverse compared with the others by the analysis of HPLC.

Table S2. Solvent effect on the enantioselectivity. [a]

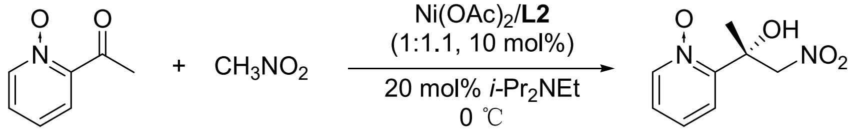

Entry	Slovent	Yield (%)[b]	ee (%)[c]
1	THF	99	91
2	EtOH	99	81
3	CH_2Cl_2	73	83
4	AcOEt	88	83
5	Toluene	88	85
6	CH_3CN	92	86
7	2-Me-THF	98	90

[a] Reactions were carried out with 2-acylpyridine *N*-oxides (0.2 mmol) with *i*-Pr_2NEt (20 mol%) in a mixture of solvent (0.8 mL) and CH_3NO_2 (0.2 mL) for 20 h. [b] Isolated yield. [c] Determined by chiral HPLC.

Table S3. Effect of substrate concentration in the asymmetric Henry reaction. [a]

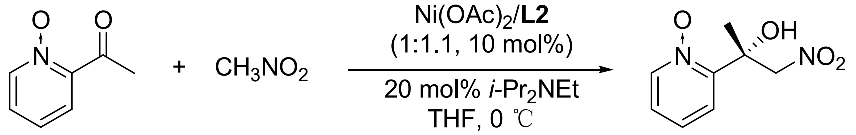

Entry	[**1a**] (M)	Yield (%)[b]	ee (%)[c]
1	0.2	99	91
2	0.4	99	84
3	0.1	89	89

[a] Reactions were carried out with 2-acylpyridine *N*-oxides (0.2 mmol) with *i*-Pr_2NEt (20 mol%) in a mixture of THF (0.3, 0.8 or 1.8 mL) and CH_3NO_2 (0.2 mL) for 20 h. [b] Isolated yield. [c] Determined by chiral HPLC.

Table S4. Screening of the amount of nitromethane in the asymmetric Henry reaction. [a]

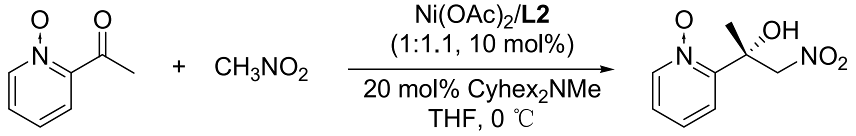

Entry	The amount of CH_3NO_2	The amount of THF	Yield (%)[b]	ee (%)[c]
1	50 μL	1.0 mL	82	77
2	0.1 mL	0.9 mL	87	92
3	0.15 mL	0.85 mL	99	86
4	0.2 mL	0.8 mL	99	94
5	0.3 mL	0.7 mL	99	89

[a] Reactions were carried out with 2-acylpyridine *N*-oxides (0.2 mmol) with *N,N*-dicyclohexylmethylamine (20 mol%) in a mixture of THF and CH_3NO_2 for 20 h. [b] Isolated yield. [c] Determined by chiral HPLC.

3. ESI-MS analysis of the mixture of Ni/L2/1a

(a)

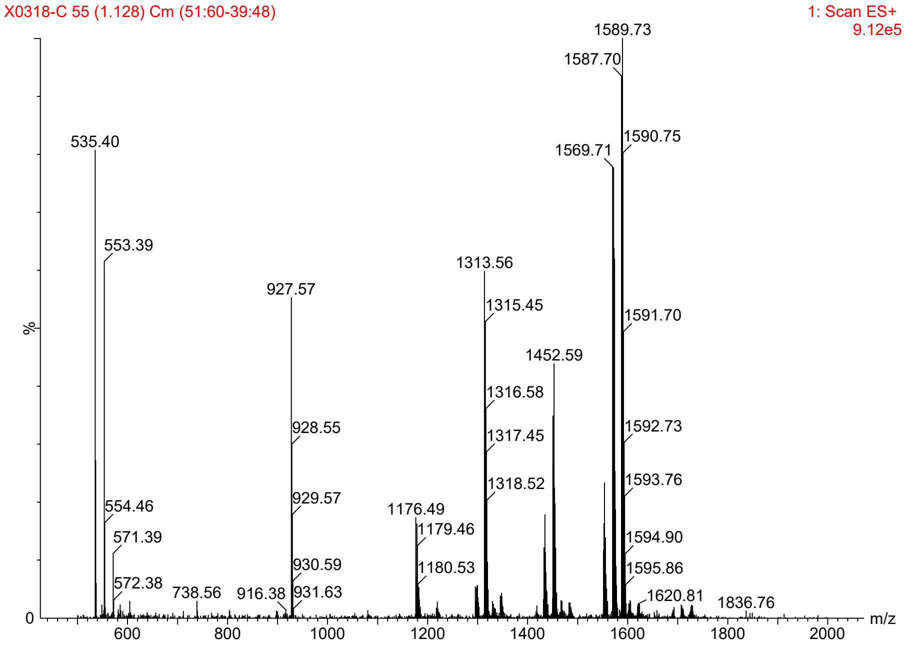

(b)

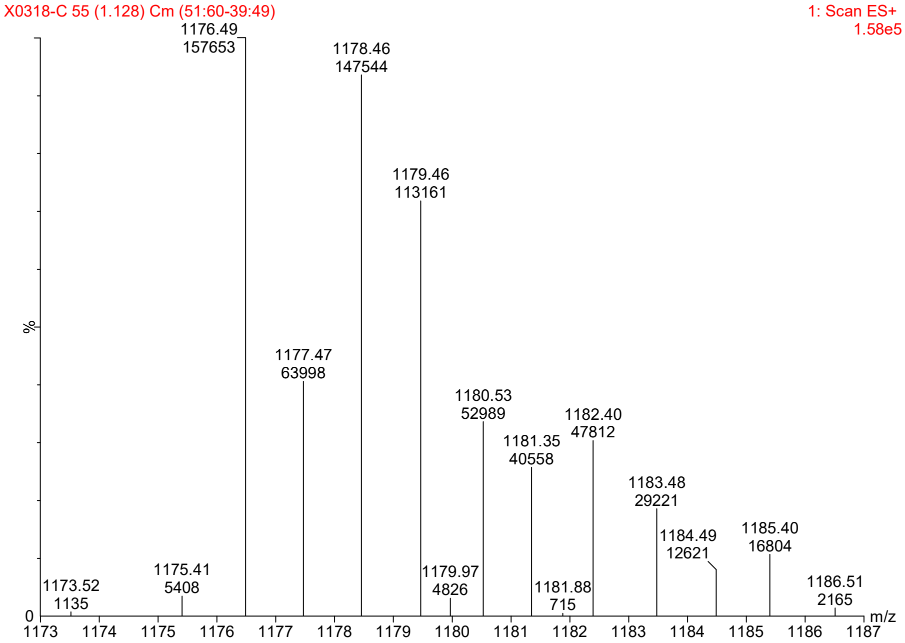

(c)

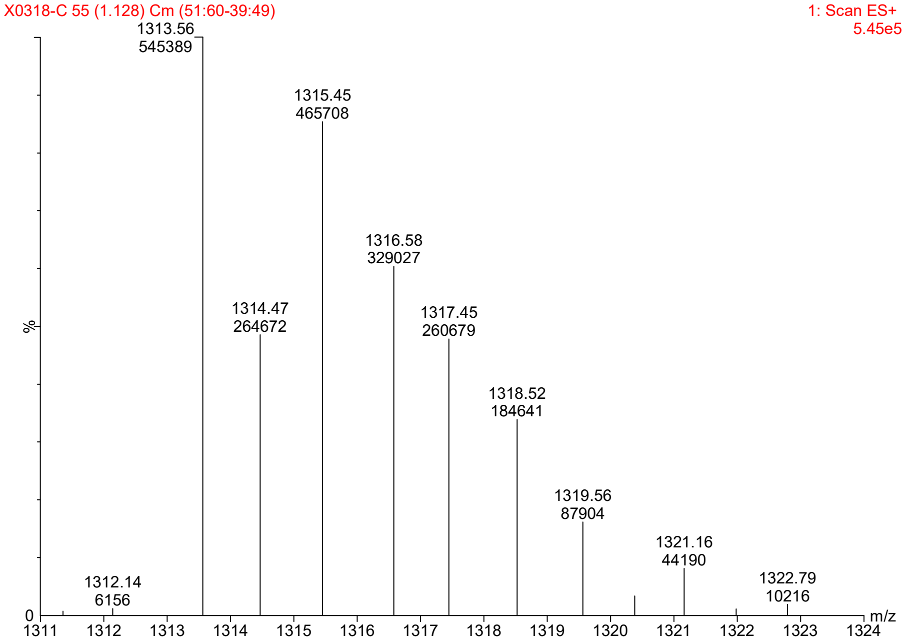
X0318-C 55 (1.128) Cm (51:60-39:49)
1: Scan ES+
5.45e5
1313.56
545389
1315.45
465708
1316.58
329027
1314.47
264672
1317.45
260679
1318.52
184641
1319.56
87904
1321.16
44190
1312.14
6156
1322.79
10216
%
0
m/z
1311 1312 1313 1314 1315 1316 1317 1318 1319 1320 1321 1322 1323 1324

(d)

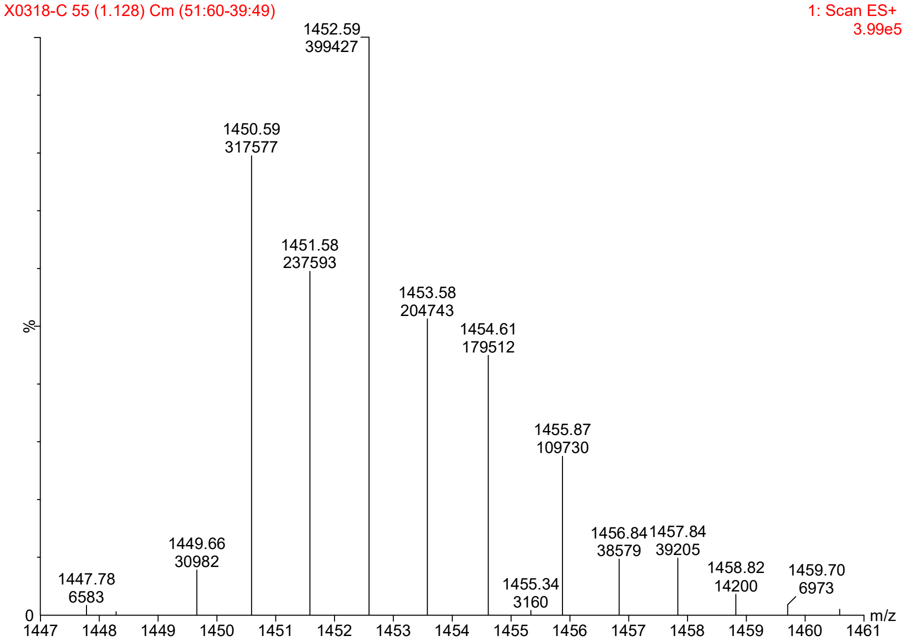
X0318-C 55 (1.128) Cm (51:60-39:49)
1: Scan ES+
3.99e5
1452.59
399427
1450.59
317577
1451.58
237593
1453.58
204743
1454.61
179512
1455.87
109730
1456.84
38579
1457.84
39205
1449.66
30982
1447.78
6583
1455.34
3160
1458.82
14200
1459.70
6973
%
0
m/z
1447 1448 1449 1450 1451 1452 1453 1454 1455 1456 1457 1458 1459 1460 1461

(e)

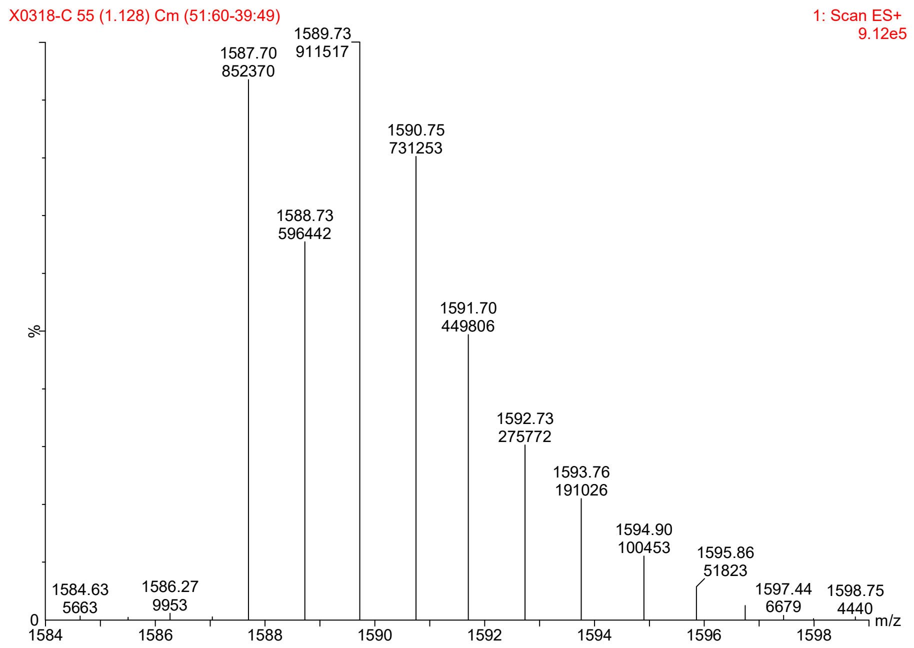

Calcd. For $C_{58}H_{55}N_7NiO_{13}S_2$ $[M_{L2} – H + NiOAc + M_{1a}]^+$: 1179.26; Found: 1179.46

Calcd. For $C_{65}H_{62}N_8NiO_{15}S_2$ $[M_{L2} – H + NiOAc + 2M_{1a}]^+$: 1316.31; Found: 1316.58

Calcd. For $C_{72}H_{69}N_9NiO_{17}S_2$ $[M_{L2} – H + NiOAc + 3M_{1a}]^+$: 1453.36; Found: 1453.58

Calcd. For $C_{79}H_{76}N_{10}NiO_{19}S_2$ $[M_{L2} – H + NiOAc + 4M_{1a}]^+$: 1590.41; Found: 1590.75

Figure S1. ESI-MS of $Ni(OAc)_2$/**L2**/**1a** = 0.1/0.11/1 (**a**–**e**).

(f)

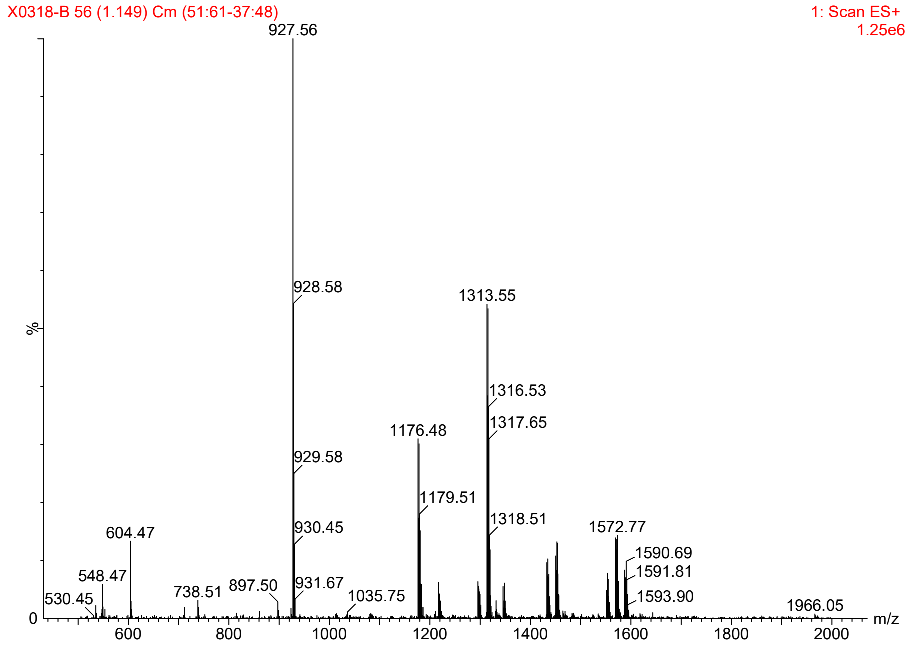

Figure S2. ESI-MS of $Ni(OAc)_2$/**L2**/**1a** = 1/1.1/1 (f).

4. HPLC chromatograms for ee determination

(1) **2a**

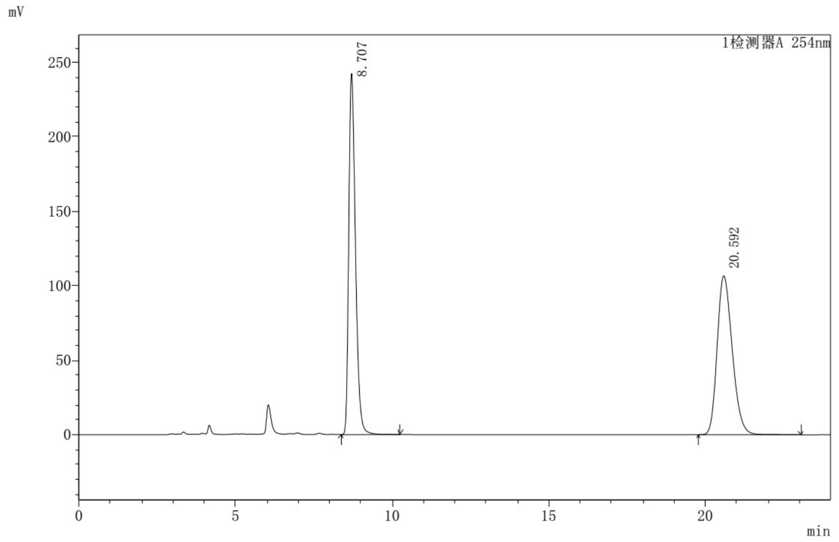

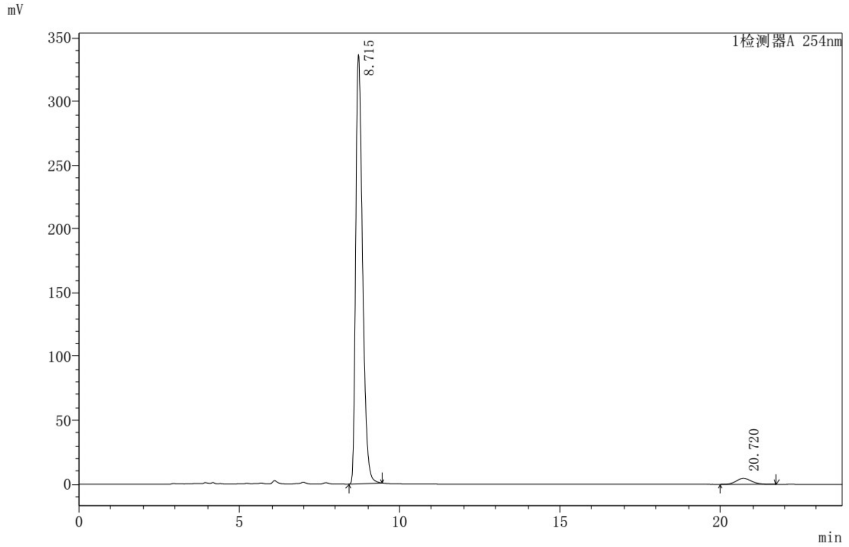

Peak#	Retention Time	Area%
1	8.715	97.041
2	20.720	2.959
Total		100.000

(2) **2b**

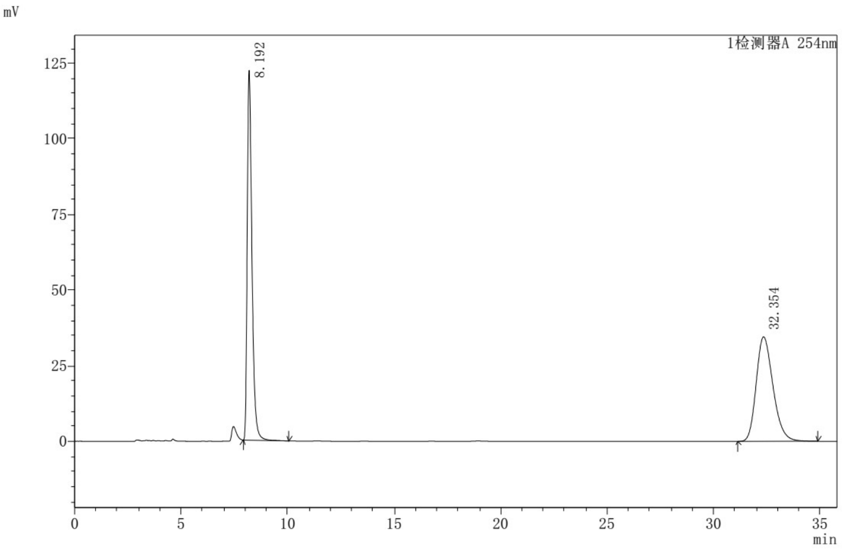

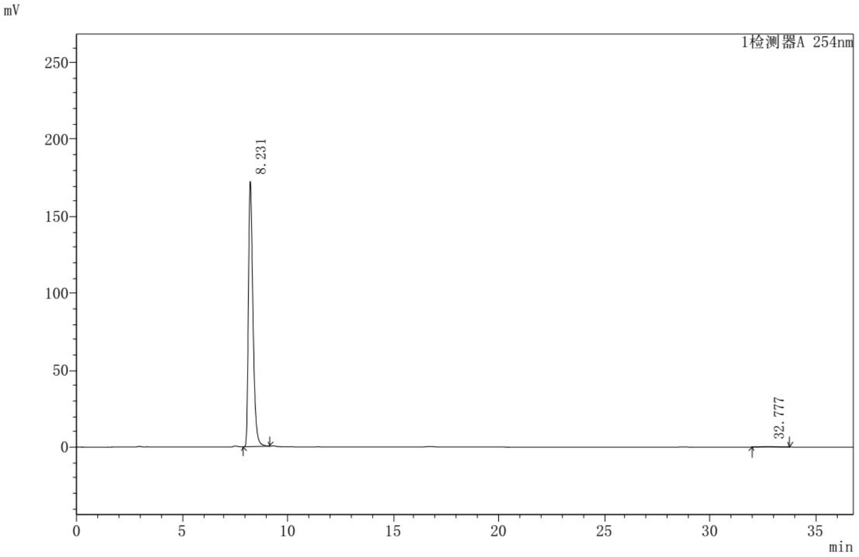

Peak#	Retention Time	Area%
1	8.231	99.336
2	32.777	0.664
Total		100.000

(3) **2c**

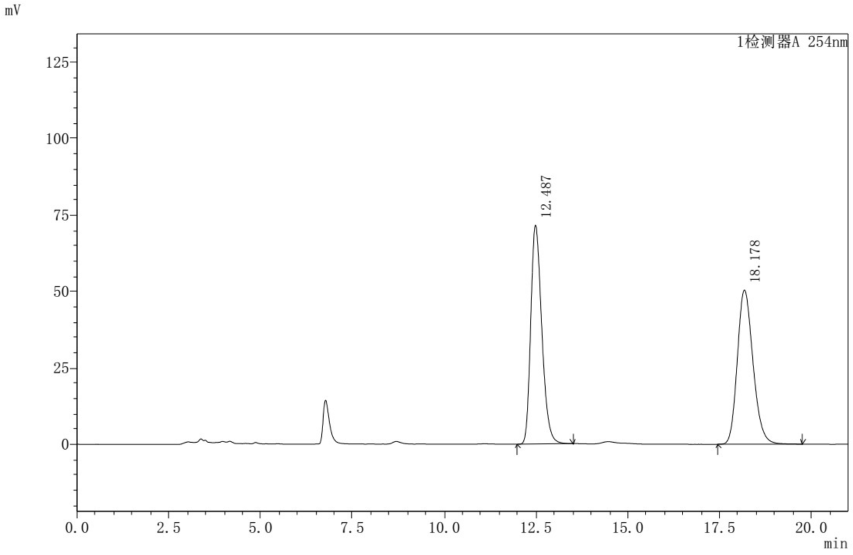

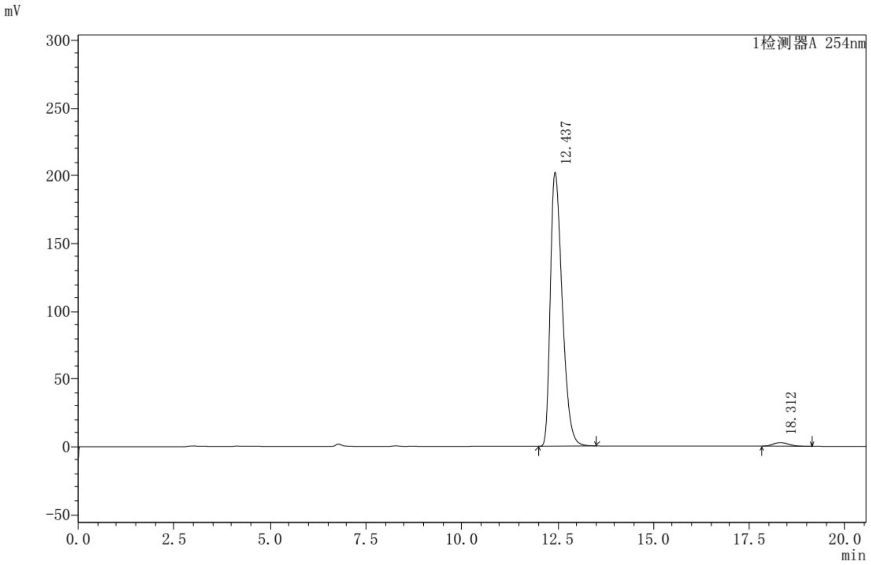

Peak#	Retention Time	Area%
1	12.437	98.264
2	18.312	1.736
Total		100.000

(4) **2d**

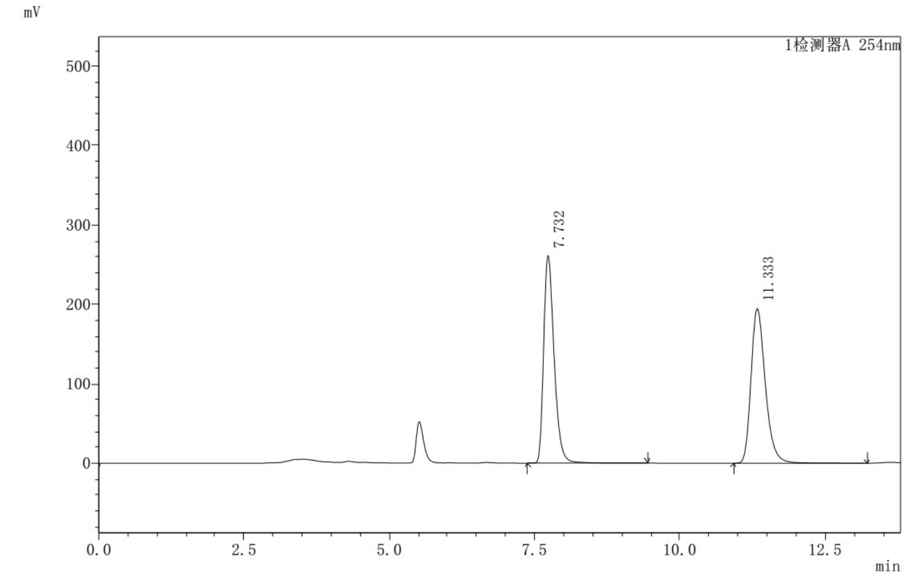

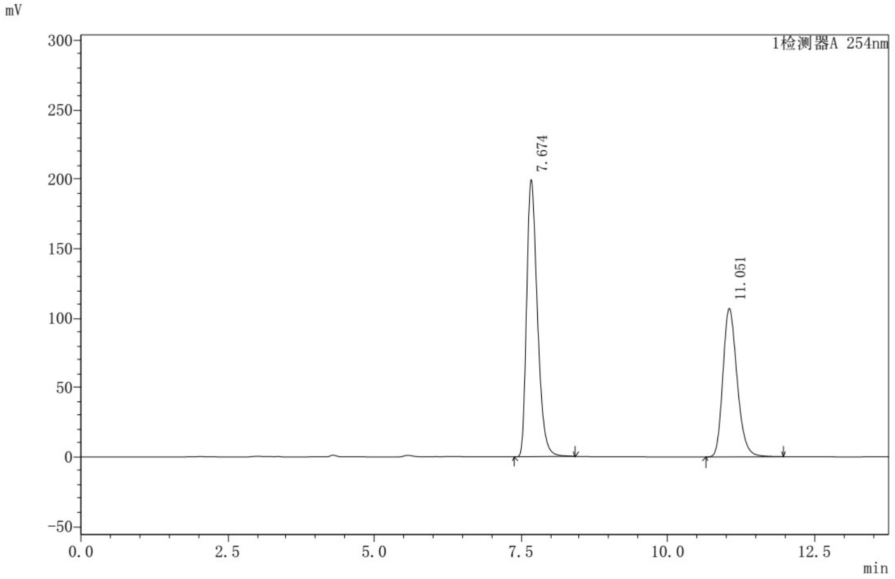

Peak#	Retention Time	Area%
1	7.674	58.543
2	11.051	41.457
Total		100.000

(5) **2e**

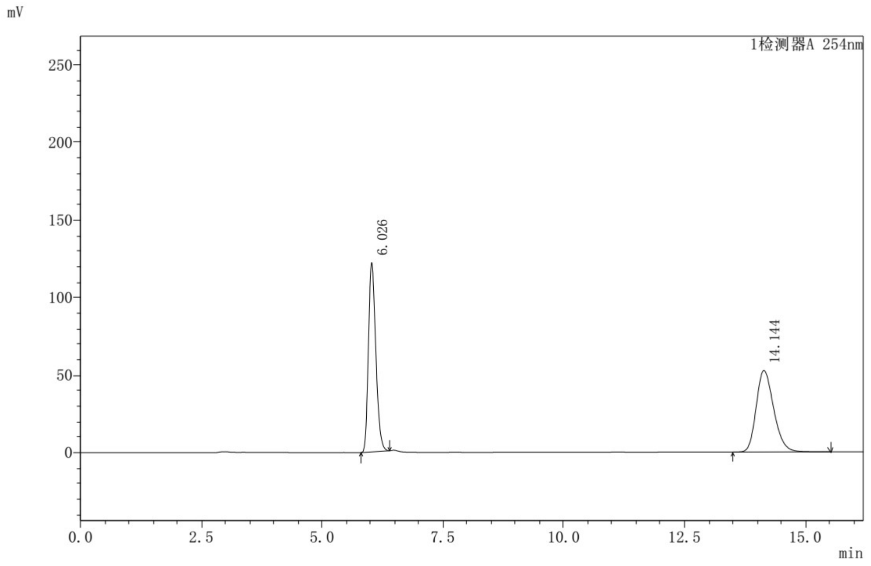

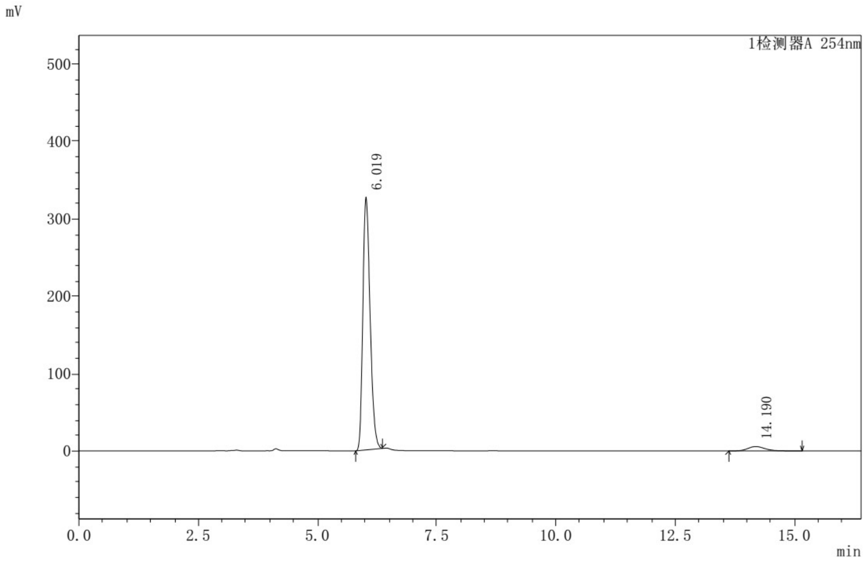

Peak#	Retention Time	Area%
1	6.019	96.093
2	14.190	3.907
Total		100.000

(6) **2f**

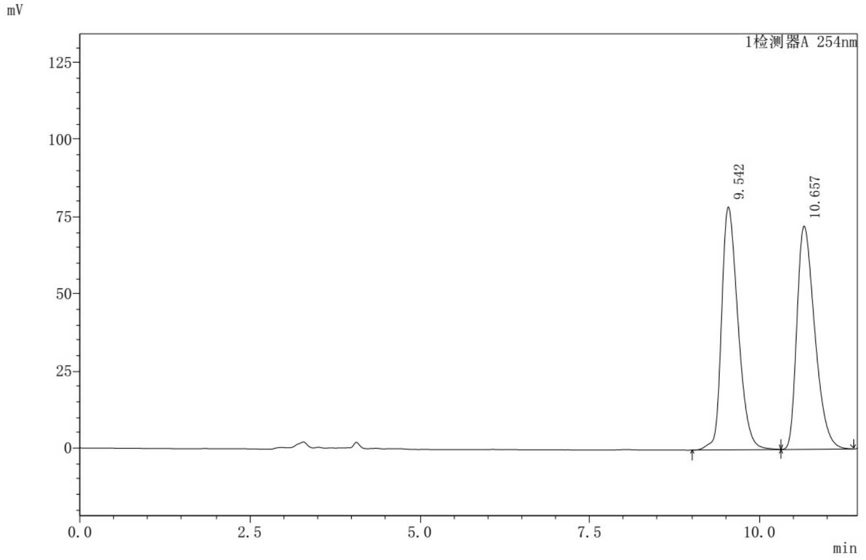

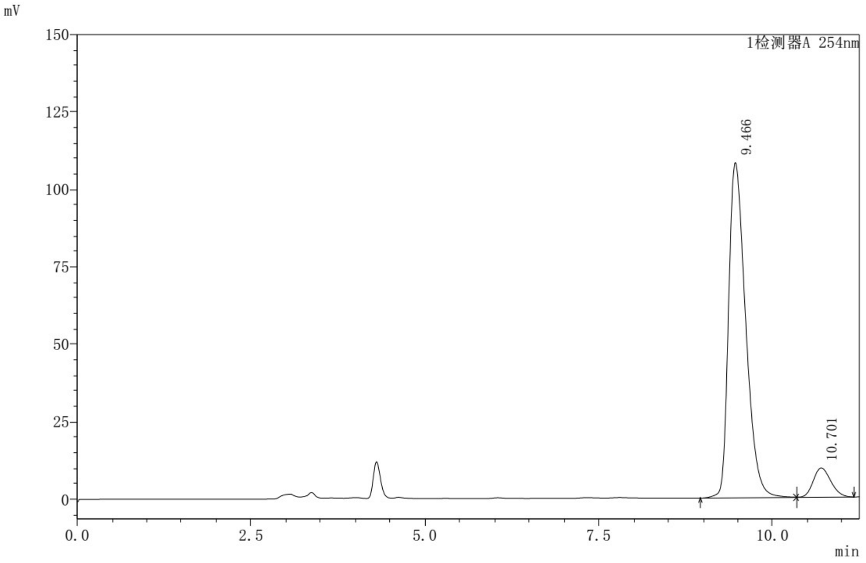

Peak#	Retention Time	Area%
1	9.466	91.725
2	10.701	8.275
Total		100.000

(7) **2g**

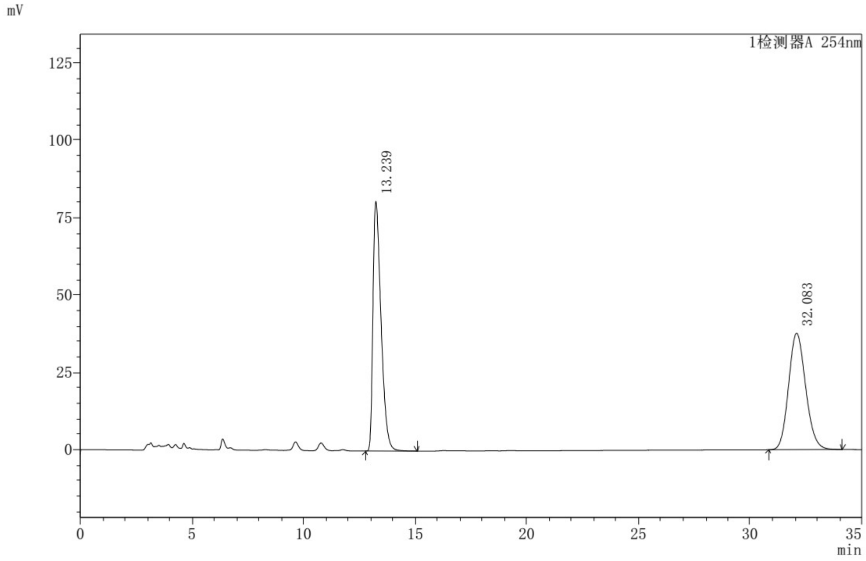

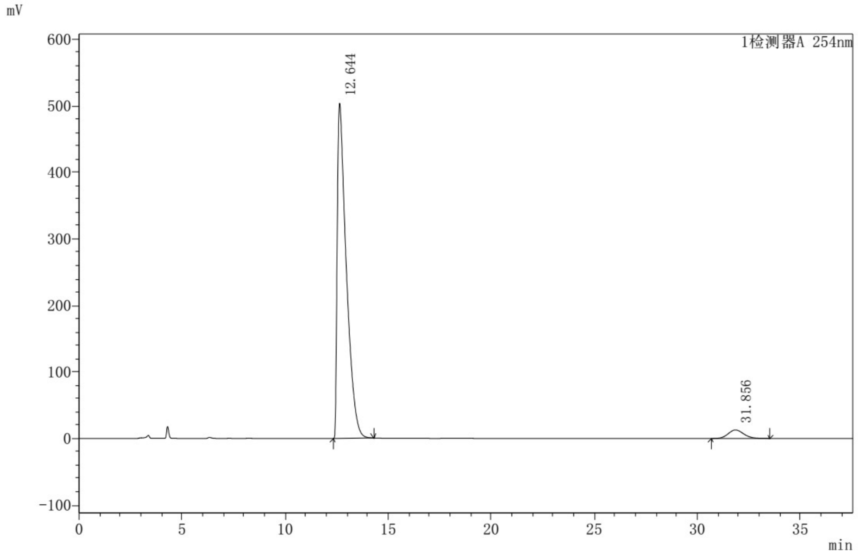

Peak#	Retention Time	Area%
1	12.644	95.848
2	31.856	4.152
Total		100.000

(8) **2h**

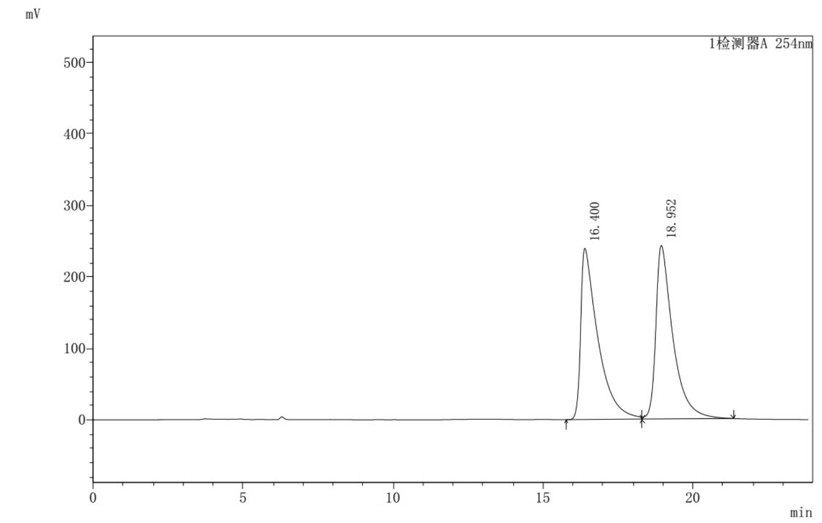

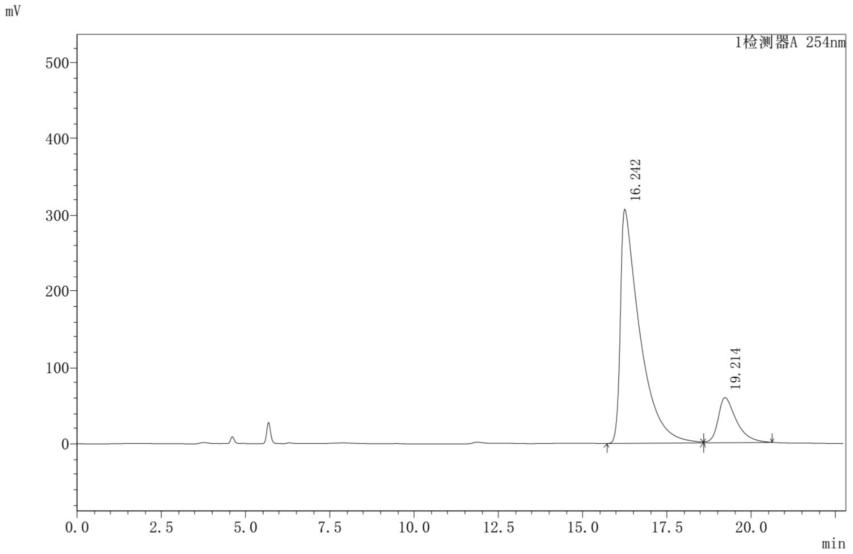

Peak#	Retention Time	Area%
1	16.242	84.636
2	19.214	15.364
Total		100.000

(9) **2i**

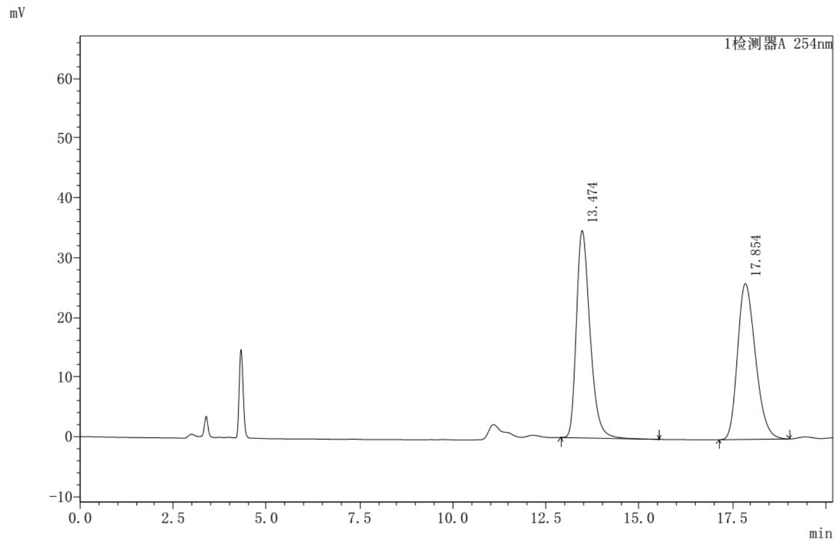

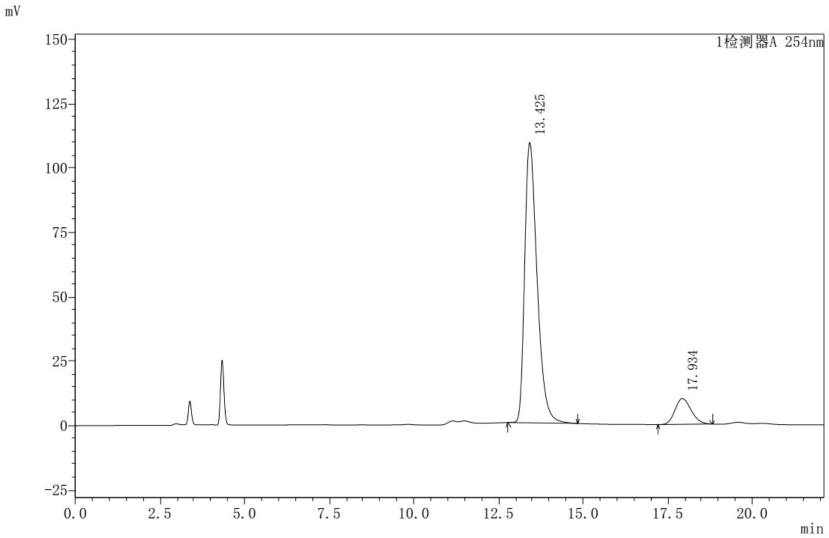

Peak#	Retention Time	Area%
1	13.425	89.302
2	17.934	10.698
Total		100.000

5. Copies of NMR spectra

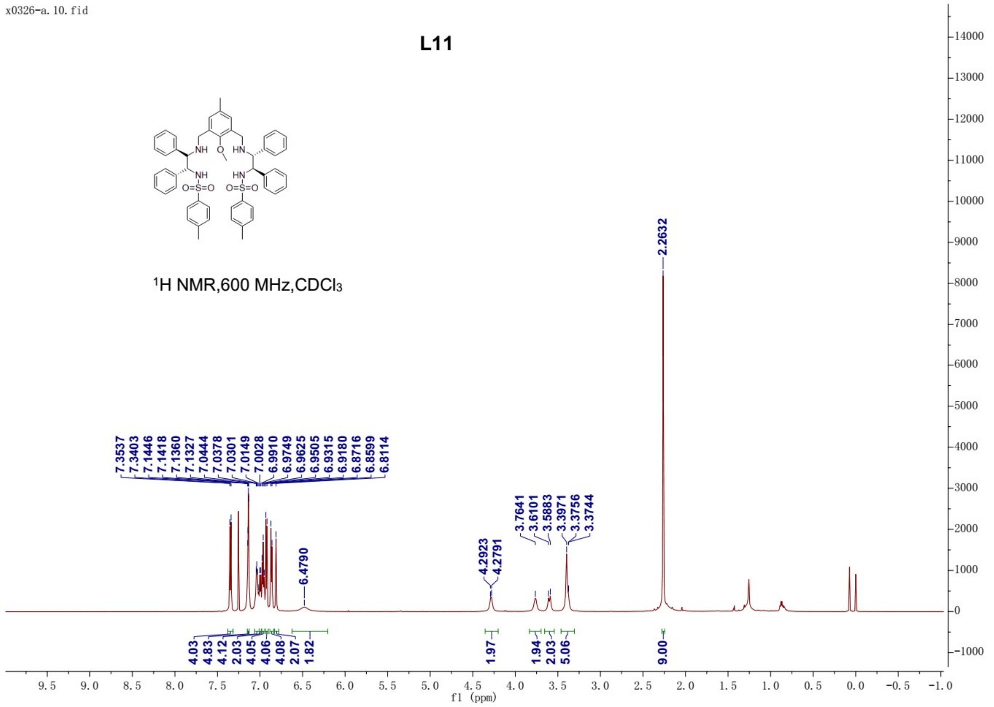

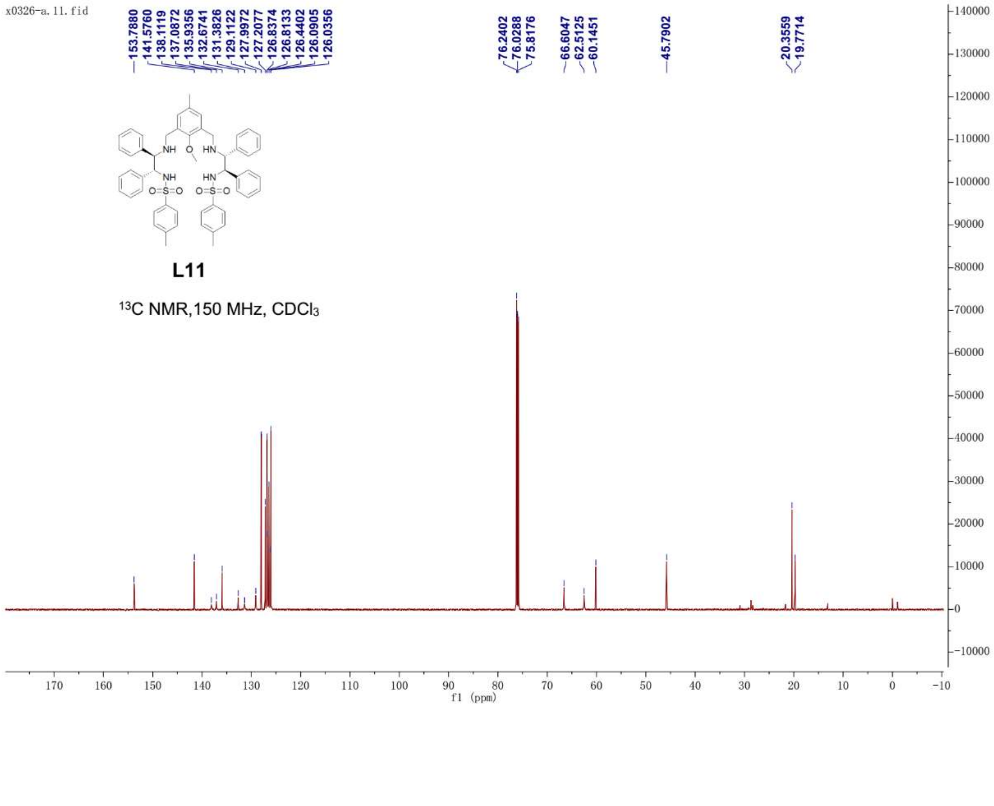

2a

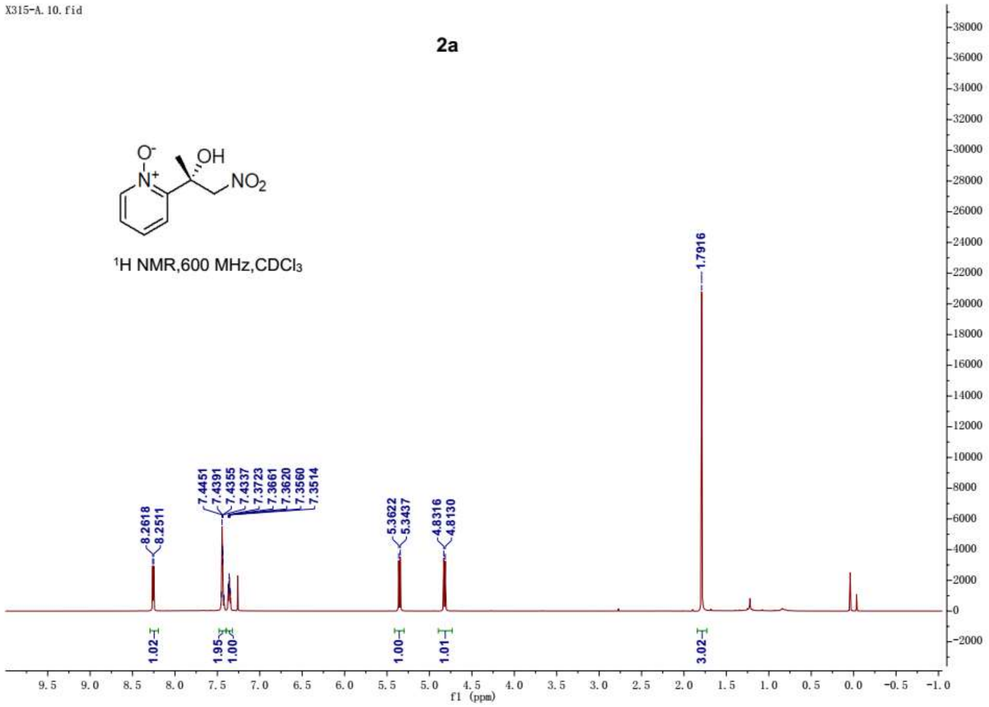
X315-A.10.fid
^{1}H NMR,600 MHz,CDCl$_3$
8.2618
8.2511
7.4451
7.4391
7.4355
7.4337
7.3723
7.3661
7.3620
7.3560
7.3514
5.3622
5.3437
4.8316
4.8130
1.7916
1.02
1.95
1.00
1.00
1.01
3.02
f1 (ppm)

2b

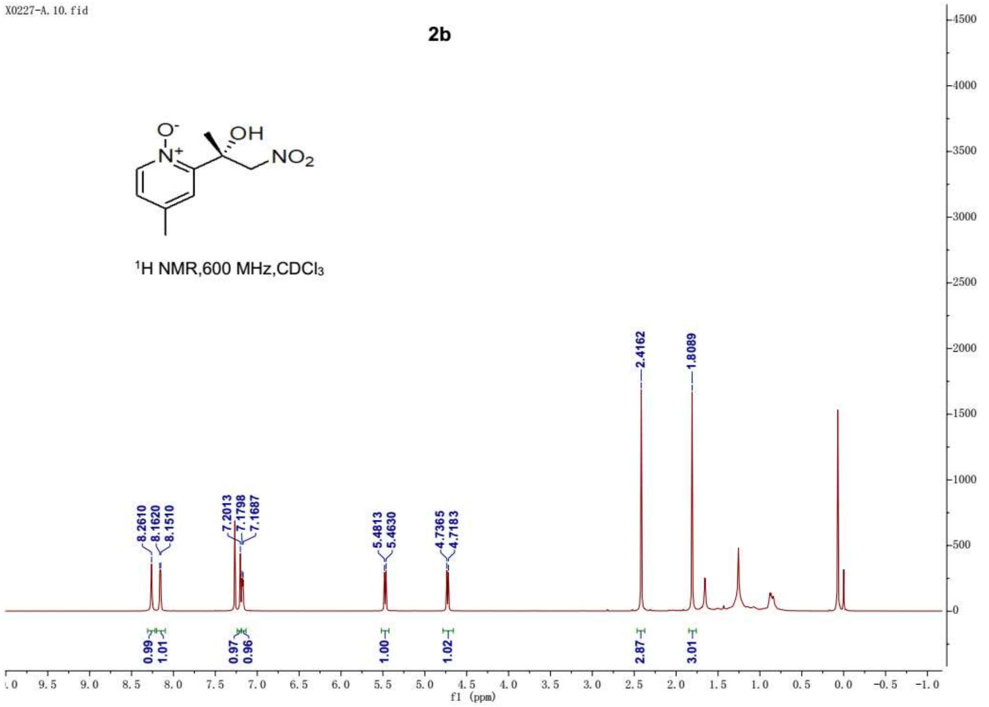
X0227-A.10.fid
^{1}H NMR,600 MHz,CDCl$_3$
8.2610
8.1620
8.1510
7.2013
7.1798
7.1687
5.4813
5.4630
4.7365
4.7183
2.4162
1.8089
0.99
1.01
0.97
0.96
1.00
1.02
2.87
3.01
f1 (ppm)

X0227-B.10.fid

2c

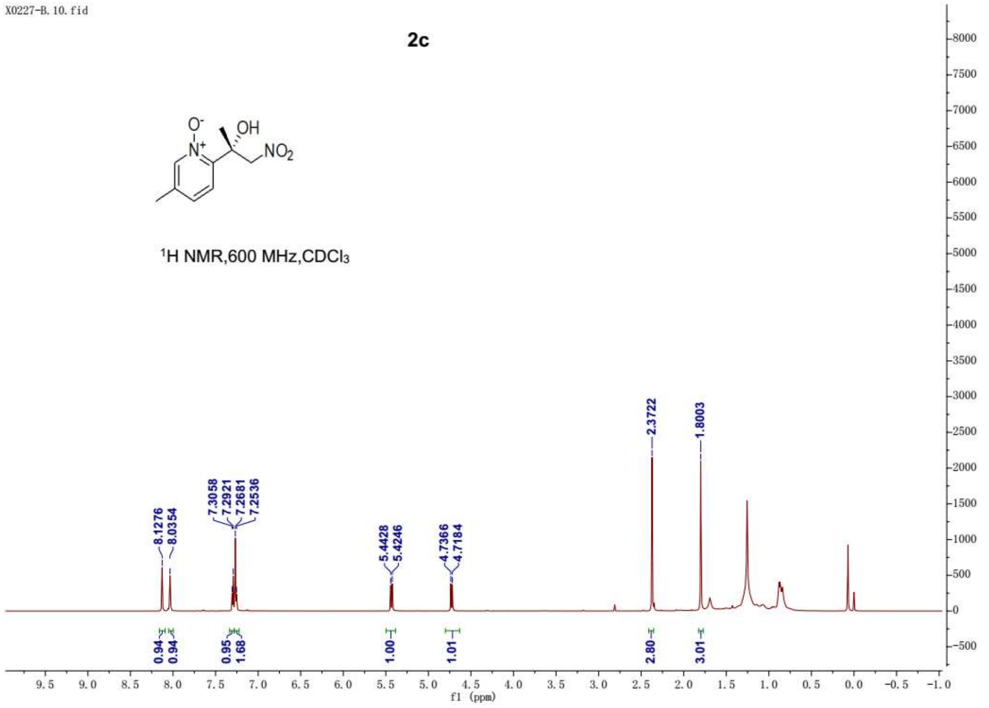

X027-C.10.fid

2d

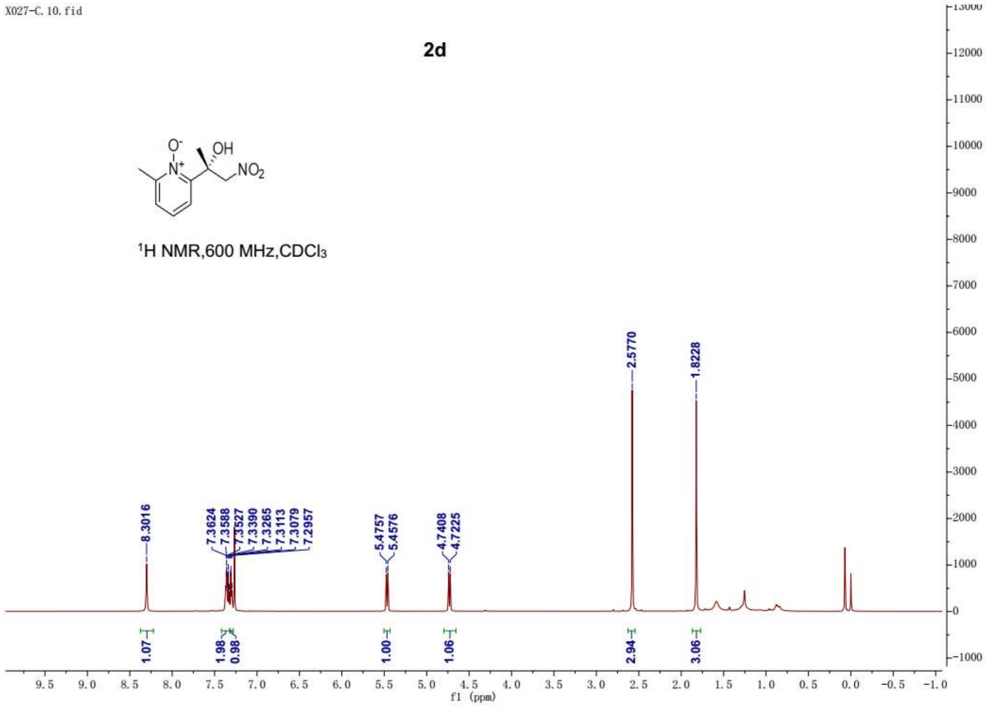

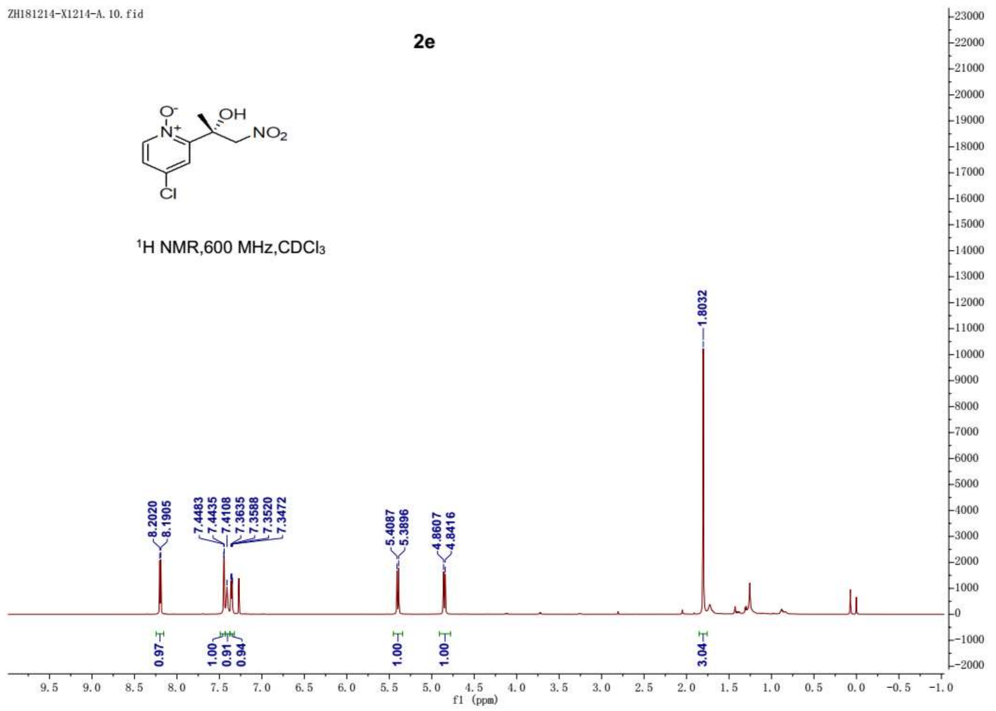
ZH181214-X1214-A.10.fid
2e
^{1}H NMR,600 MHz,$CDCl_3$
8.2020
8.1905
7.4483
7.4435
7.4108
7.3635
7.3588
7.3520
7.3472
5.4087
5.3896
4.8607
4.8416
1.8032
0.97
1.00
0.91
0.94
1.00
1.00
3.04
f1 (ppm)

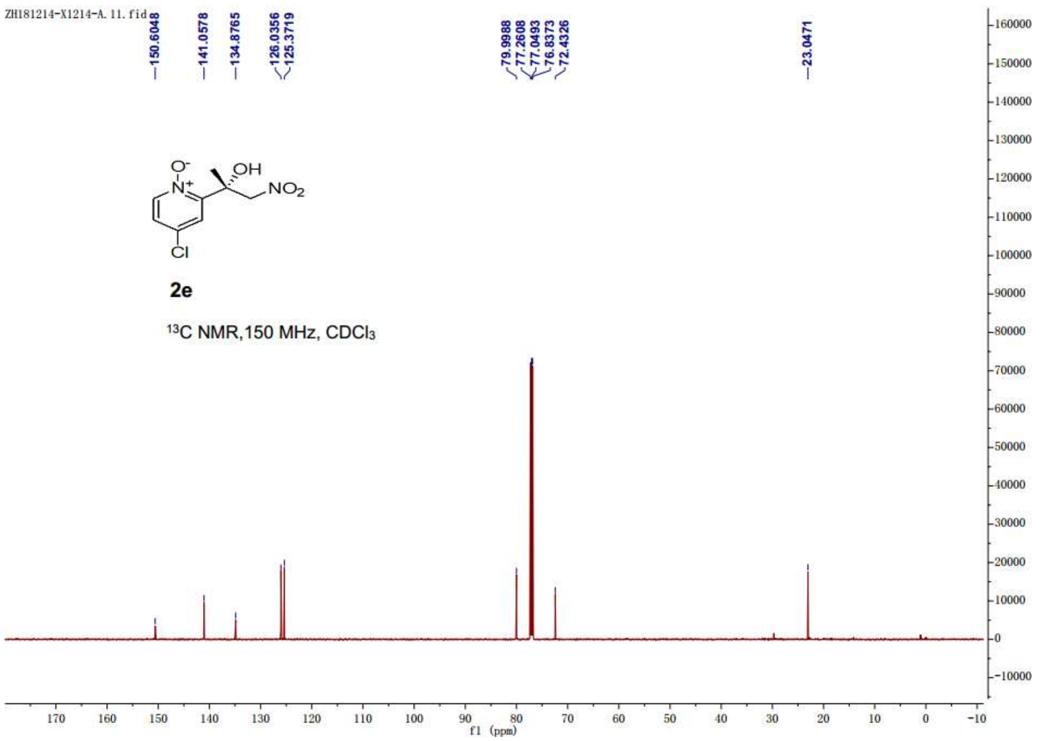
ZH181214-X1214-A.11.fid
150.6048
141.0578
134.8765
126.0356
125.3719
79.9988
77.2608
77.0493
76.8373
72.4326
23.0471
2e
^{13}C NMR,150 MHz, $CDCl_3$
f1 (ppm)

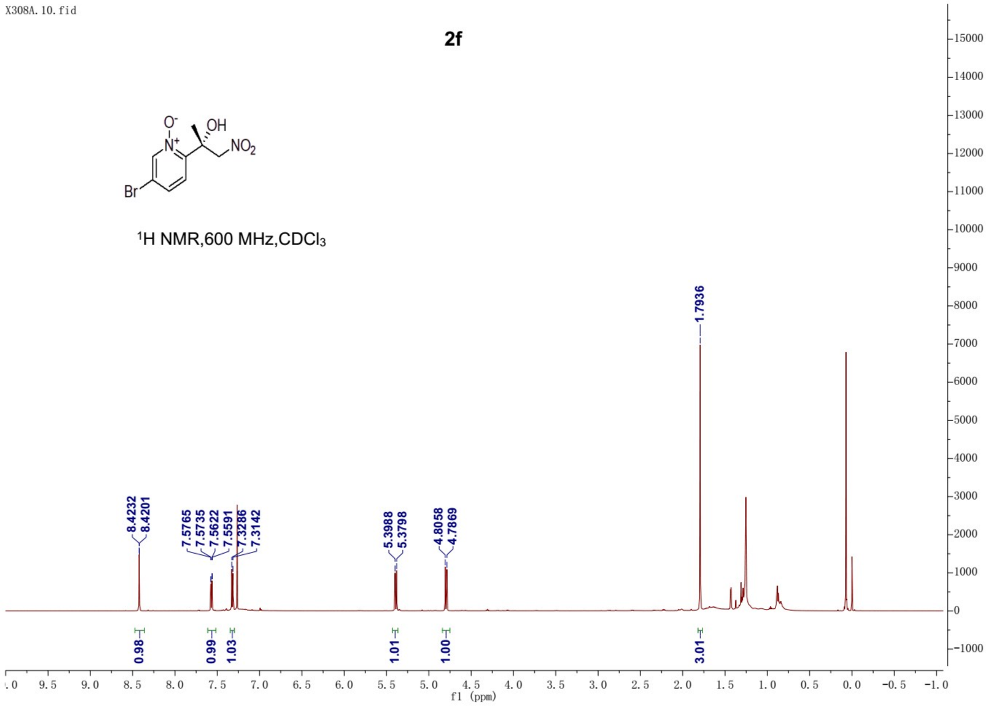
X308A.10.fid
2f
O-
N+
OH
NO2
Br
1H NMR,600 MHz,CDCl3
8.4232
8.4201
7.5765
7.5735
7.5622
7.5591
7.3286
7.3142
5.3988
5.3798
4.8058
4.7869
1.7936
0.98
0.99
1.03
1.01
1.00
3.01
f1 (ppm)

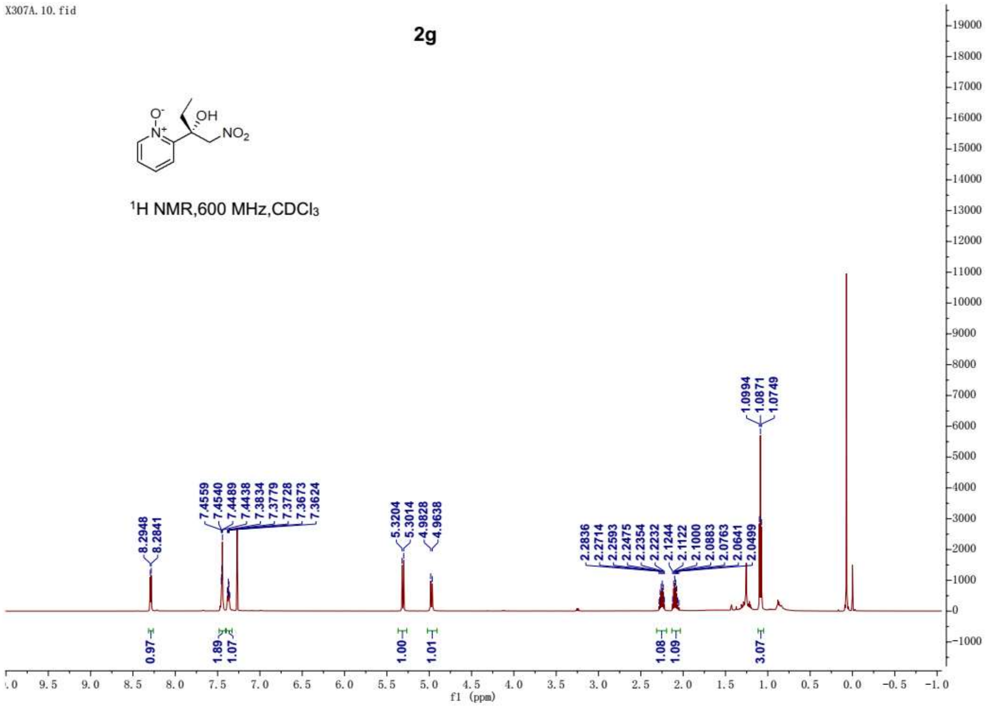
X307A.10.fid
2g
O-
N+
OH
NO2
1H NMR,600 MHz,CDCl3
8.2948
8.2841
7.4559
7.4540
7.4489
7.4438
7.3834
7.3779
7.3728
7.3673
7.3624
5.3204
5.3014
4.9828
4.9638
2.2836
2.2714
2.2593
2.2475
2.2354
2.2232
2.1244
2.1122
2.1000
2.0883
2.0763
2.0641
2.0499
1.0994
1.0871
1.0749
0.97
1.89
1.07
1.00
1.01
1.08
1.09
3.07
f1 (ppm)

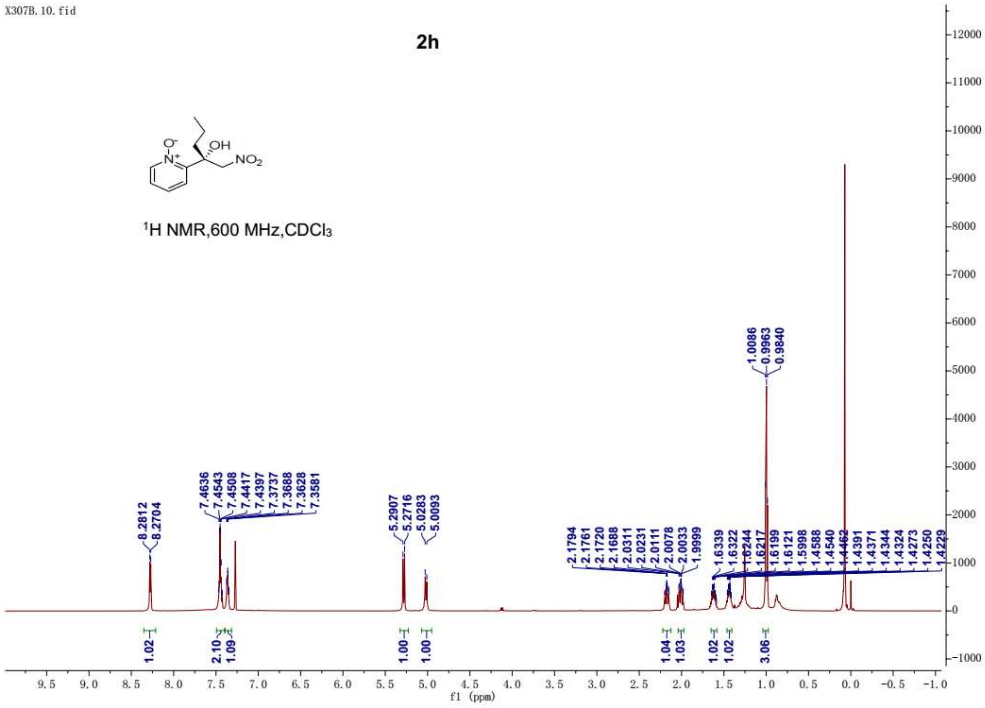
X307B.10.fid
2h
^{1}H NMR,600 MHz,CDCl$_3$
8.2812 8.2704
7.4636 7.4543 7.4508 7.4417 7.4397 7.3737 7.3688 7.3628 7.3581
5.2907 5.2716 5.0283 5.0093
2.1794 2.1761 2.1720 2.1688 2.0311 2.0231 2.0111 2.0078 2.0033 1.9999
1.6339 1.6322 1.6244 1.6217 1.6199 1.6121 1.5998 1.4588 1.4540 1.4462 1.4391 1.4371 1.4344 1.4324 1.4273 1.4250 1.4229
1.0086 0.9963 0.9840
1.02 2.10 1.09 1.00 1.00 1.04 1.03 1.02 1.02 3.06
f1 (ppm)

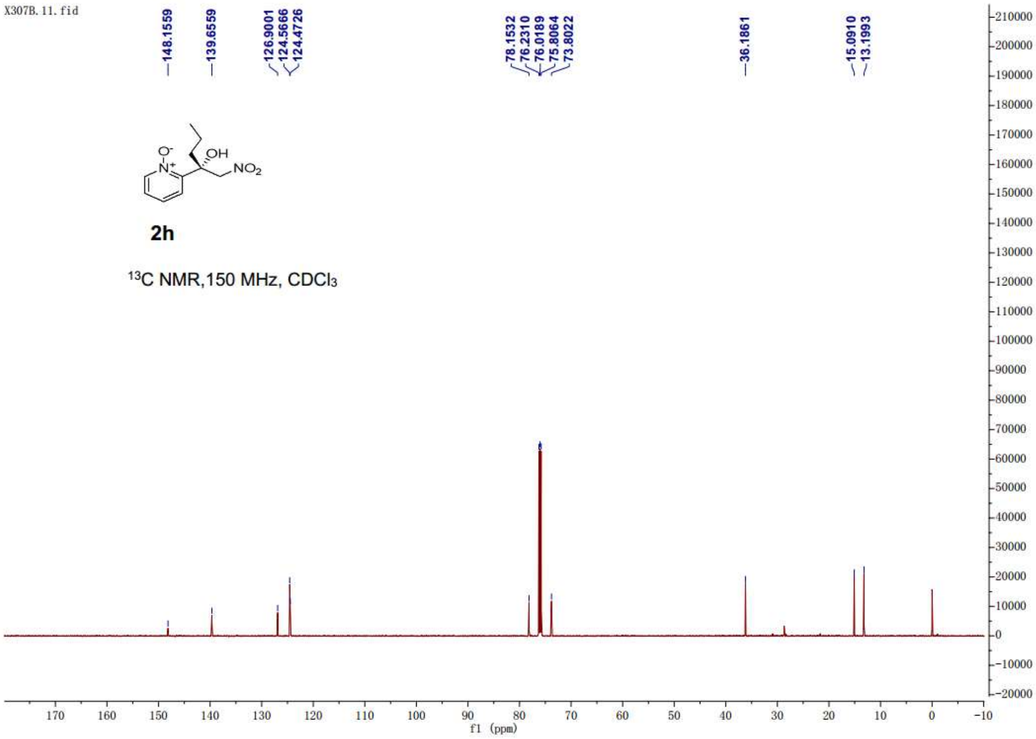
X307B.11.fid
148.1559
139.6559
126.9001 124.5666 124.4726
78.1532 76.2310 76.0189 75.8064 73.8022
36.1861
15.0910 13.1993
2h
^{13}C NMR,150 MHz, CDCl$_3$
f1 (ppm)

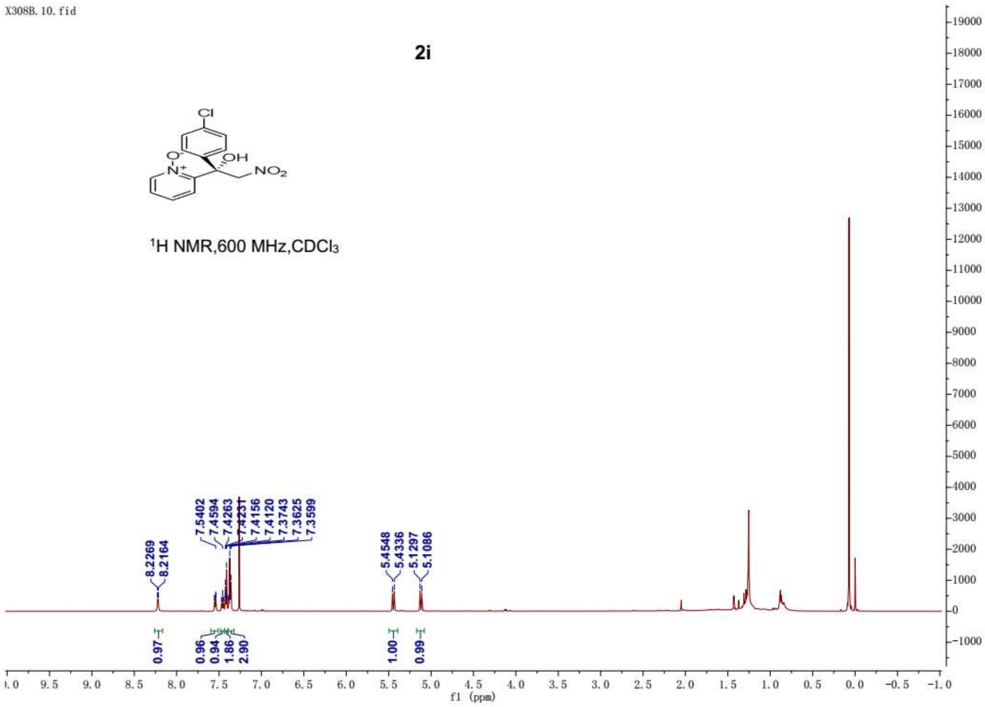
X308B.10.fid
2i
Cl
OH
NO2
1H NMR,600 MHz,CDCl3
8.2269
8.2164
7.5402
7.4594
7.4263
7.4231
7.4156
7.4120
7.3743
7.3625
7.3599
5.4548
5.4336
5.1297
5.1086
0.97
0.96
0.94
1.86
2.90
1.00
0.99
f1 (ppm)
